# Correction: *In vivo* modulation of ubiquitin chains by *N*-methylated non-proteinogenic cyclic peptides

**DOI:** 10.1039/d1cb90015c

**Published:** 2021-05-11

**Authors:** Joseph M. Rogers, Mickal Nawatha, Betsegaw Lemma, Ganga B. Vamisetti, Ido Livneh, Uri Barash, Israel Vlodavsky, Aaron Ciechanover, David Fushman, Hiroaki Suga, Ashraf Brik

**Affiliations:** Department of Chemistry, Graduate School of Science, The University of Tokyo 7-3-1 Hongo, Bunkyo-ku Tokyo 113-0033 Japan hsuga@chem.s.u-tokyo.ac.jp; Department of Drug Design and Pharmacology, University of Copenhagen Copenhagen 2100 Denmark; Schulich Faculty of Chemistry, Technion-Israel Institute of Technology Haifa 3200008 Israel abrik@technion.ac.il; Department of Chemistry and Biochemistry, Center for Biomolecular Structure and Organization, University of Maryland College Park MD 20742 USA; The Rappaport Faculty of Medicine and Research Institute, Technion-Israel Institute of Technology Haifa 31096 Israel

## Abstract

Correction for ‘*In vivo* modulation of ubiquitin chains by *N*-methylated non-proteinogenic cyclic peptides’ by Joseph M. Rogers *et al.*, *RSC Chem. Biol.*, 2021, **2**, 513–522, DOI: 10.1039/D0CB00179A.

The author regrets that the funding information was incorrectly shown in the acknowledgements section of the original manuscript.

This study was also funded by the National Institutes of Health under grant GM065334 to D.F. and A.B.

The Royal Society of Chemistry apologises for these errors and any consequent inconvenience to authors and readers.

## Supplementary Material

